# The direction of effects between parenting and adolescent affective well-being in everyday life is family specific

**DOI:** 10.1038/s41598-023-43294-5

**Published:** 2023-09-26

**Authors:** Savannah Boele, Anne Bülow, Adriene M. Beltz, Amaranta de Haan, Jaap. J. A. Denissen, Loes Keijsers

**Affiliations:** 1 Department of Psychology, Education and Child Studies, Erasmus University Rotterdam, P.O. Box 1738, 3000 DR Rotterdam, The Netherlands; 2 Department of Psychology, University of Michigan, Ann Arbor, USA; 3 Department of Developmental Psychology, Utrecht University, Utrecht, The Netherlands

**Keywords:** Human behaviour, Psychology

## Abstract

Numerous theories and empirical studies have suggested that parents and their adolescent children reciprocally influence each other. As most studies have focused on group-level patterns, however, it remained unclear whether this was true for every family. To investigate potential heterogeneity in directionality, we applied a novel idiographic approach to examine the effects between parenting and adolescent well-being in each family separately. For 100 days, 159 Dutch adolescents (*M*_age_ = 13.31, 62% female) reported on affective well-being and four parenting dimensions. The family-specific effects of pre-registered (https://osf.io/7n2jx/) dynamic structural equation models indeed revealed that a reciprocal day-to-day association between parenting and adolescent affective well-being was present only in some families, with the proportion of families displaying a reciprocal association varying across the four parenting dimensions (11–55%). In other families, either parenting predicted the adolescent’s affective well-being (8–43%) or vice versa (10–27%), or no day-to-day associations were found (16–60%). Adolescents with higher trait levels of environmental sensitivity and neuroticism were more strongly affected by parenting. Thus, findings suggest that the ways in which parents and adolescents influence each other in everyday life are unique, stressing the need to move towards an idiographic parenting science.

## Introduction

A long-standing question in parenting research has been the direction of effects: Who influences whom (the most)?^1,2^. Typically, this question has been studied by asking follow-up questions, such as: Is the parent mainly affecting their adolescent child? Or is the adolescent the most active agent and driving changes in parenting? Or are influences equal, with parents and adolescents reciprocally affecting each other? Reciprocity in the parent-adolescent relationship is now an established concept in many theories^2–4^, but could this contemporary theoretical consensus be inaccurate—at least for some families?^5^.

**Figure 1:**
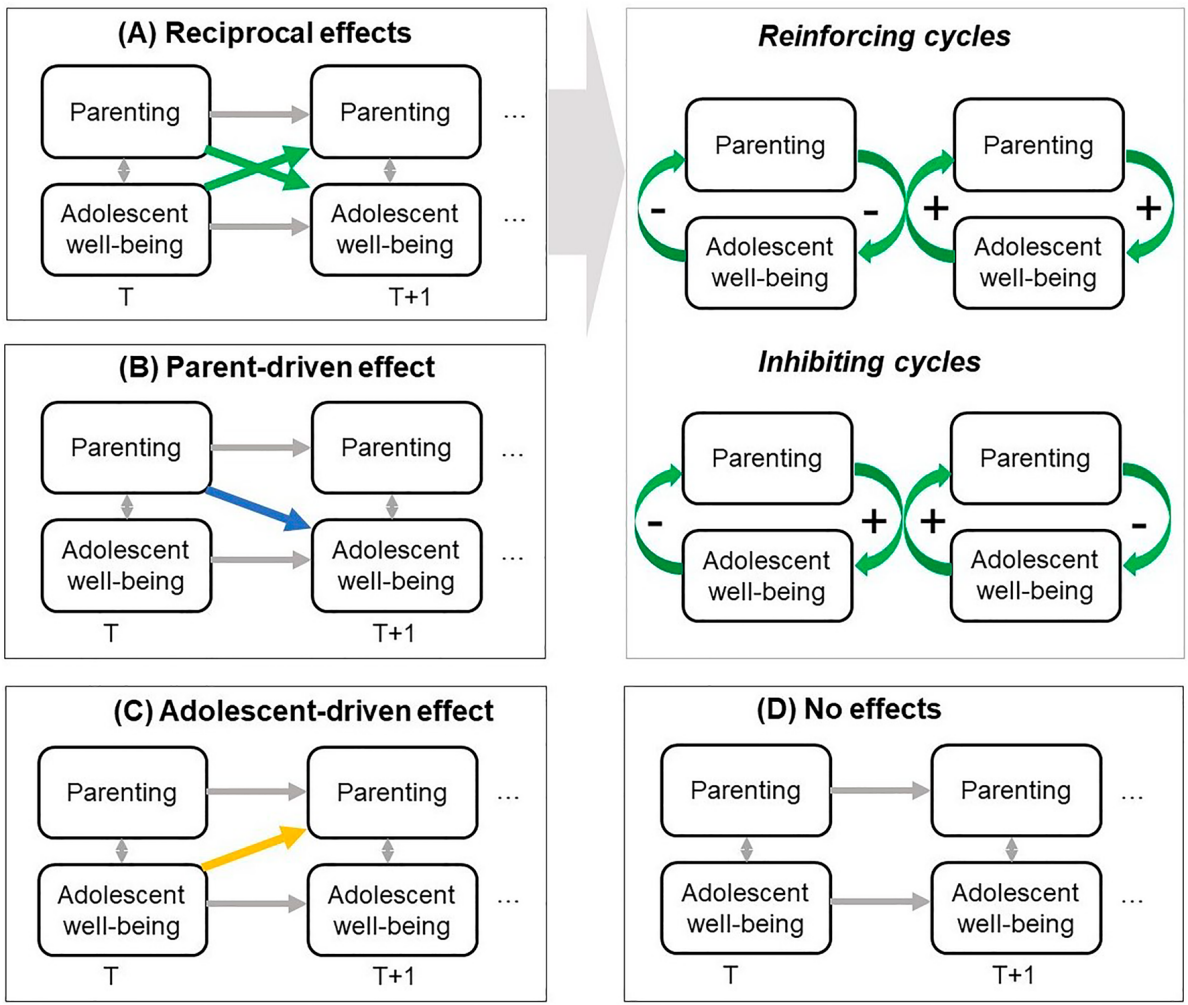
Theoretical different directions of effects between parenting and adolescent well-being within a family. (**A**) Reciprocal effects: fluctuations in parenting and adolescent well-being predict each other from one time point (e.g., day) to the next. These reciprocal effects can differ in nature, such that reinforcing and inhibitory cycles are possible, including positive (+) and/or negative effects (−). (**B**) Parent-driven effect: fluctuations in parenting predict later adolescent well-being but not vice versa. (**C**) Adolescent-driven effect: fluctuations in adolescent well-being predict later parenting but not vice versa. (**D**) No effects: fluctuations in parenting and adolescent well-being do not predict each other over time.

Although theories have stated that influences within the family may be inherently reciprocal^3^, there are also theoretical notions and empirical studies suggesting that the direction of influence might differ from family to family. For example, theories on environmental sensitivity posit that people vary in their responsiveness to contextual influences, including the behavior of others^6,7^. This idea is supported by various empirical studies showing that individuals with higher trait levels of environmental sensitivity (i.e., ability to perceive, process, and respond to stimuli) and neuroticism (i.e., tendency to experience and inability to cope with negative emotions) respond more strongly to interpersonal experiences^6,8,9,29^ Additionally, adolescent girls are believed to be more sensitive to interpersonal experiences than adolescent boys^10^. Furthermore, studies have shown that some adolescents reject their parents’ authority, leading to disobedience and possibly non-responsiveness to parental demands^11^, and theories suggest that controlling and supportive parenting might only be effective in promoting adolescent well-being if such styles align with the (developmental) needs of the adolescent^12^. Thus, it is likely that parent-adolescent dyads are differentially responsive to each other and might be (a) characterized by reciprocal influences in some families, (b) largely driven by parental influences in other families (parent-driven), (c) largely driven by adolescent influences in still other families (adolescent-driven), and (d) occasionally non-existent (see Fig. 1).

### Heterogeneity in the nature of reciprocal dynamics

Even among families with reciprocal parent-adolescent influences, it is possible that the nature of these influences varies. To illustrate, when an adolescent feels sad, a parent in one family may make the adolescent feel better by providing more affection, whereas a parent in another family may instead (unintentionally) amplify the adolescent’s negative feelings by showing less affection, as the parent themselves withdraws from the expressed sadness. In dynamic systems and related theories, such phenomena are called inhibiting and reinforcing processes, respectively^13^. An inhibiting process is the driving force behind maintaining stability, whereas a reinforcing process may trigger a change. As these processes are meaningful, it is important to examine potential heterogeneity in the nature of reciprocal parent-adolescent dynamics.

### Methodological advancements: from group-level patterns to the dynamics of individual families

Most empirical parenting studies employ nomothetic methods to establish general principles^14,15^, often by examining group-level patterns^16,17^. Such examinations have indicated, for example, that adolescents whose parents display higher levels of support have better psychological well-being (e.g., fewer depressive symptoms)—on average—than adolescents whose parents display lower levels of support^18,19^. Meta-analytic work on group-level associations highlights these bidirectional associations between parenting and adolescent well-being^18,19^, which initially seems to support theoretical notions of reciprocal parent-adolescent influences^3^. At the same time, however, it has been increasingly purported that group-level patterns do not necessarily align with dynamic processes that unfold within individual families^14,20^. That is, relations between *average* parenting and *average* adolescent well-being may not describe *family-specific* relations between parenting and well-being, especially if these processes are expectedly heterogeneous across families^20,21^. Therefore, the heavy reliance on nomothetic methods in parenting science^17^, and in other fields of psychological science^22,23^, is problematic for the accuracy and implementation of scientific findings. Group-level patterns suggesting reciprocal associations between parenting and adolescent well-being^18,19,24^ could obscure the direction of the effects at the level of the individual family. Additionally, translating group-level patterns into nomothetic parenting advice might unintentionally harm families if they are not described well by the group average. Thus, to promote the well-being of adolescents^25^, there is an urgent need to gain empirical insights into how parents and adolescents impact each other within individual families. This may ultimately help practitioners develop and apply interventions tailored to a family’s dynamics and needs.

To gain these insights into how heterogeneous parents and adolescents influence each other within *individual* families, an idiographic approach is needed^14,26^. One increasingly popular method, but lacking in parenting research^17,27^, is to use intensive longitudinal data (e.g., experience sampling or daily diary data). The very first idiographic parenting studies provided evidence that parenting effects on adolescent well-being indeed varies from family to family in both magnitude^28^ and in nature (i.e., positive or negative effect)^29,30^. For instance, some adolescents benefited from supportive parenting, whereas others did not respond to it or were even negatively impacted by it. Heterogeneity in parent-driven *and* adolescent-driven effects (see Fig. 1) has not yet gained much scientific attention, though, leaving important questions unanswered: How heterogeneous is the direction of effects?

### The present study

In the present idiographic study, 159 Dutch families were meticulously followed up for 100 consecutive days to investigate the *family-specific* day-to-day dynamics between perceived parenting and adolescent affective well-being. The main aim was to test a pre-registered hypothesis (https://osf.io/7n2jx/) that some families would show reciprocal effects, whereas others would show either a parent-driven effect, an adolescent-driven effect, or no effects at all (see Fig. 1). This hypothesis was tested across eight distinct parenting-affect associations: four key dimensions of parenting with two dimensions of adolescent affective well-being. We explored whether families showed a similar direction of effects across those distinct associations and whether heterogeneity in directionality could be explained by attributes of the adolescent (i.e., demographic factors and personality traits). Furthermore, we examined whether families showed qualitatively different reciprocal effects (i.e., inhibiting and reinforcing cycles).

## Results

### Descriptive statistics and correlations

**Figure 2:**
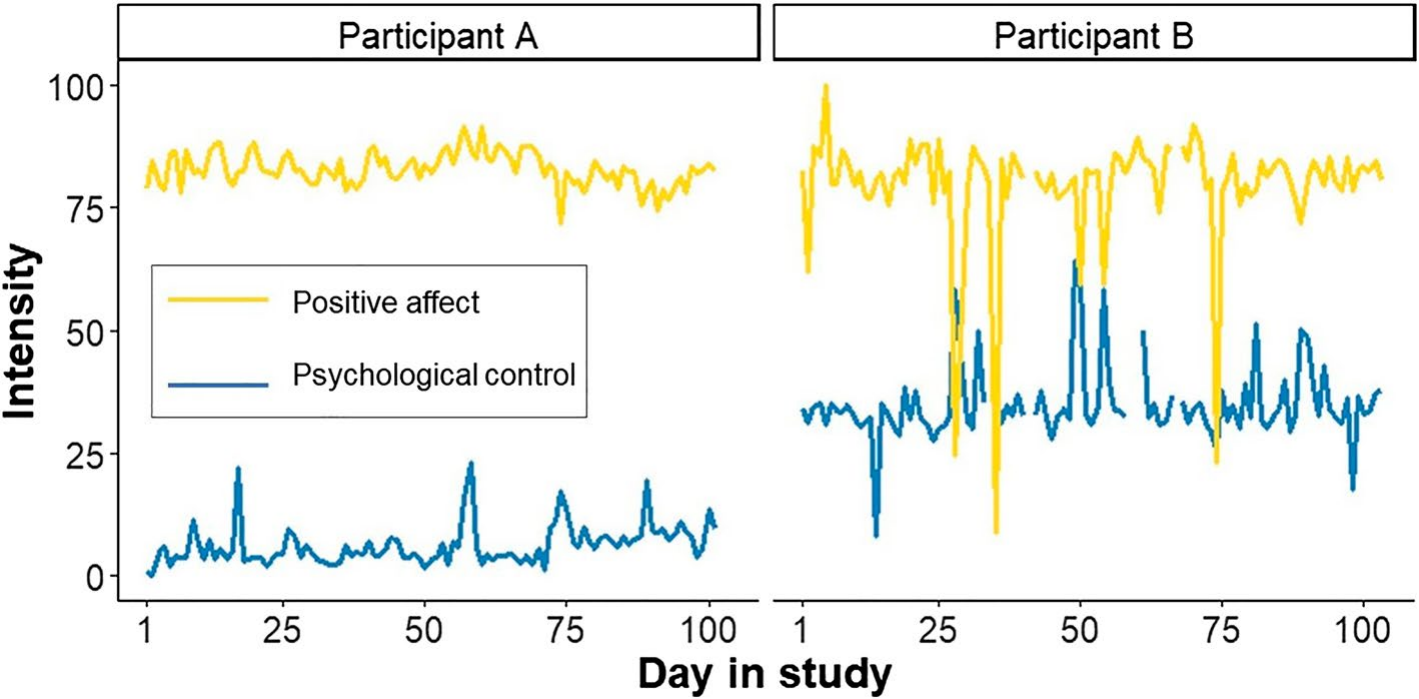
Daily fluctuations in parental psychological control and adolescent positive affect reported by two participating adolescents. Timeseries of two participants, including their daily mean scores on their level of positive affect and perceived parental psychological control across 100 days. Response scale ranged from 0 to 100.

Both perceived parenting and adolescent affect fluctuated from one day to the next (for two examples, see Fig. 2). The intraclass correlations (ICCs) for the parenting variables indicated that 56% to 67% of the variance was due to stable between-family differences and 33% to 44% due to daily fluctuations within families. For adolescent positive and negative affect, 62% and 47% of the variance was due to stable between-family differences, respectively, and the remaining 38% to 53% was due to daily fluctuations within families (Table 1).

**Table 1:** Descriptive statistics and correlations (N = 159).

	Correlations					
	1	2	3	4	5	6
1. Psychological control	–	.29**	− .27**	− .34**	− .13**	.17**
2. Behavioral control	.51**	–	− .11**	− .17**	− .03	.10**
3. Autonomy support	− .40**	− .27**	–	.39**	.20**	− .14**
4. Warmth	− .45**	− .30**	.69**	–	.33**	− .23**
5. Positive affect	− .16	− .17*	.41**	.51**	–	− .50**
6. Negative affect	.41**	.37**	− .30**	− .38**	− .66**	–
*M*	6.69	16.89	83.31	74.57	76.49	10.99
*SD*	11.92	20.94	17.39	24.97	20.68	14.94
ICC	.61	.67	.56	.59	.62	.47
*T*_total_	14,516	14,512	14,520	14,531	14,819	14,784

*M* = sample mean. *SD* = standard deviation. ICC = intraclass correlation coefficient. *T* = number of observations. All the items ranged from 0 to 100. Correlations at the within-family level are presented above the diagonal, and at the between-family level are under the diagonal.

**p < .001, *p < .05.

Most parenting dimensions correlated weakly with adolescent affect at the within-family level (*r*s between − .23 and .20; see Table 1), with the exception of the moderate correlation between parental warmth and adolescent positive affect (*r* = .33, *p* < .001). These within-family correlations indicate that, on average, adolescents reported more parental psychological control on days when they experienced less positive affect (*r* = − .13, *p* < .001) and more negative affect (*r* = .17, *p* < .001). More adolescent-perceived parental autonomy support and warmth co-fluctuated with more positive affect (*r*s ≥ .20, *p* < .001) and less negative affect (*r*s ≤ − .14, *p* < .001). Furthermore, adolescents reported more behavioral control on days they experienced more negative affect (*r* = .10, *p* < .001) but not less positive affect (*r* = − .03, *p* = .226). The correlations at the between-family level were similar to the within-family correlations in sign (i.e., positive or negative), but were larger in magnitude, with all between-family correlations being moderate in size (*r*s between − .38 and .41; see Table 1).

### The average daily dynamics between parenting and adolescent affect

Results from the models for positive affect had fixed effects indicating that, on average, reciprocal effects were found with parental autonomy support and warmth (see Table S1 in the Supporting Information). Specifically, increases in autonomy support and warmth predicted increased positive affect the next day (β = .05 and .09). Vice versa, increased positive affect predicted more autonomy support and warmth (βs = .07). However, fluctuations in adolescents’ positive affect were not preceded or followed by fluctuations in parental psychological or behavioral control (on average).

Results from the models for negative affect (see Supplementary Table S2) showed that, on average, there were parent-driven effects for parental psychological control and behavioral control, such that increases in psychological and behavioral control predicted more next-day negative affect within the average adolescent (β = .04 and .05). However, no significant average lagged effects were found for parental autonomy support and warmth. Although these results show the average day-to-day effects in the sample, they do not provide information on how perceived parenting and adolescents’ affective well-being are linked in each individual family.

### Family-specific effects: heterogeneity in direction of effects

In line with our hypothesis, models for both positive and negative affect revealed that the direction of effects for parenting-affect associations was heterogeneous across families. Depending on the combination of parenting and affect dimensions, 11.4% to 54.7% of families demonstrated a reciprocal effect, 8.2% to 43.4% a parent-driven effect, 10.1% to 27.0% an adolescent-driven effect, and 15.7% to 60.1% (close to) null effects (see Table 2). The family-specific estimates thus suggest that the direction of effects in day-to-day parent-adolescent dynamics varied across families and across dimensions of parenting and adolescent affective well-being.

**Table 2:** Direction of effects within families for parenting-affect associations.

Cross-lagged association	Direction of effects				Total *N*^a^
	Reciprocal *N* (%)	Parent-driven *N* (%)	Adolescent-driven *N* (%)	No effects *N* (%)	
*Positive affect*					
1. Psychological control	36 (23.2%)	53 (34.2%)^↑^	24 (15.5%)^↓^	42 (27.1%)	155
2. Behavioral control	36 (22.9%)	44 (28.0%)	26 (16.6%)^↓^	51 (32.4%)^↑^	157
3. Autonomy support	58 (36.7%)^↑^	29 (18.3%)	37 (23.4%)	34 (21.5%)	158
4. Warmth	**87 (54.7%)**^↑^	31 (19.5%)^↓^	16 (10.1%)^↓^	25 (15.7%)^↓^	159
*Negative affect*					
5. Psychological control	28 (18.1%)^↓^	15 (9.7%)^↓^	31 (20.0%)	**81 (52.3%)**^↑^	155
6. Behavioral control	28 (17.8%)^↓^	68 (43.3%)^↑^	17 (10.8%)^↓^	44 (28.0%)	157
7. Autonomy support	18 (11.4%)^↓^	24 (15.1%)^↓^	21 (13.3%)^↓^	**95 (60.1%)**^↑^	158
8. Warmth	29 (18.2%)^↓^	13 (8.2%)^↓^	43 (27.0%)	74 (46.5%)^↑^	159

The group size in bold represents the majority of the sample in the given association.

^a^A few families had no temporal variance in the parenting dimension, and therefore, had no lagged estimates for that association.

^↑^Proportion greater than expected by chance (i.e., 25%).

^↓^Proportion less than expected by chance (i.e., 25%).

**Figure 3:**
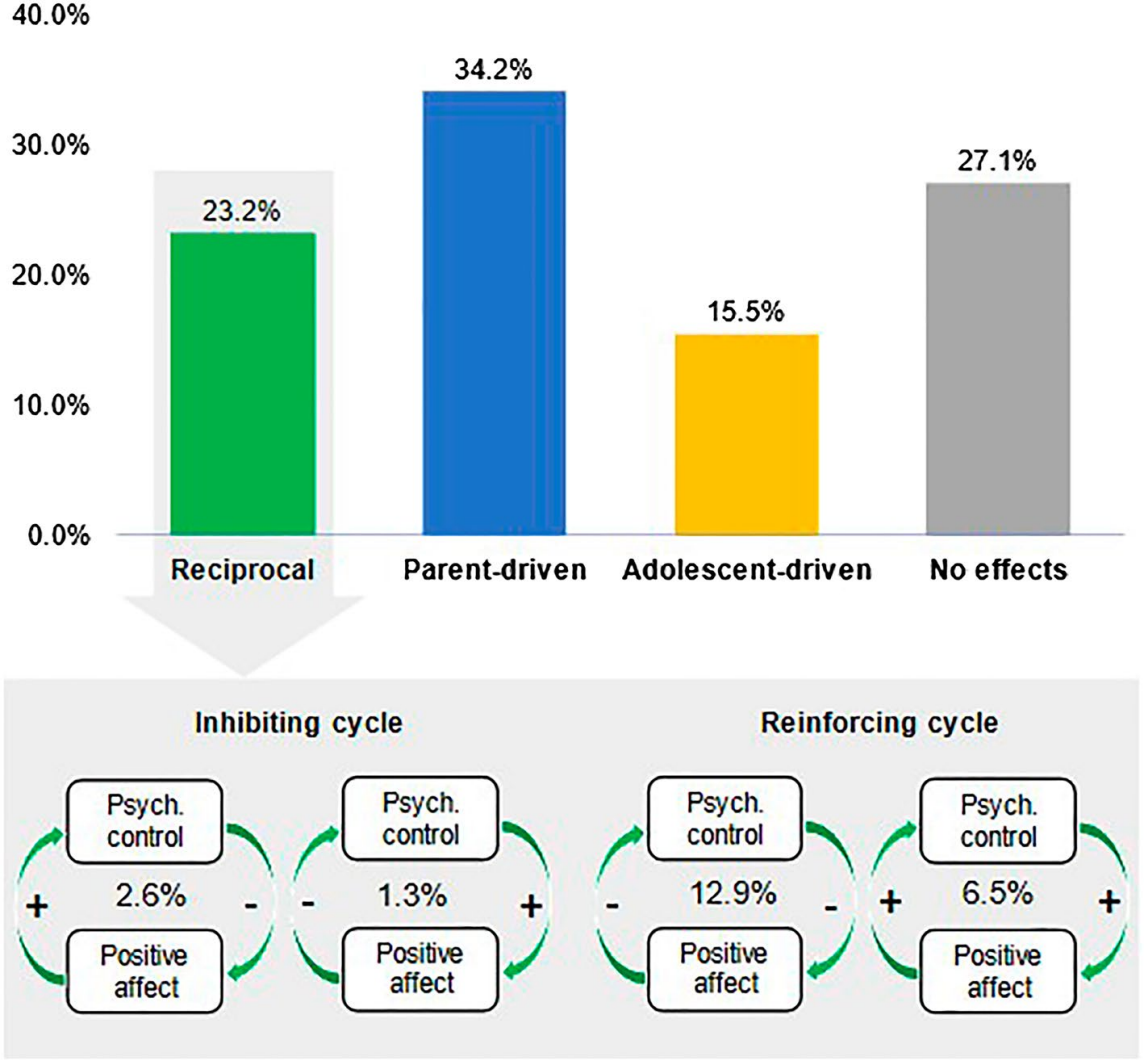
Family-specific effects for parental psychological control and adolescent positive affect. Displayed are the percentage of families (*n* = 155, see also Table 2) who showed different directions of effects for the association between parental psychological control and adolescent positive affect. Among the families with reciprocal effects (23.2%), displayed is the number who showed one of the four qualitatively different cycles. + = positive effect size. − = negative effect size.

We illustrate the heterogeneity in the direction of effects with one association (see Fig. 3). Fluctuations in parental psychological control and adolescent positive affect reciprocally predicted each other the next day in 23.2% of families (*n* = 36). In 34.2% (*n* = 53), perceived parental psychological control predicted adolescents’ positive affect the next day, but not vice versa (parent-driven effect). Conversely, in 15.5% (*n* = 24), adolescent positive affect predicted parental psychological control, but not the other way around (adolescent-driven effect). In the remaining 27.1% (*n* = 42), no day-to-day effects were found between parental psychological control and adolescents’ positive affect.

We then explored whether the proportions of families showing reciprocal, parent-driven, adolescent-driven, and null effects were different from what would be expected by chance (i.e., 25% of families showing each type of effect). Most of the proportions (22 of 32; see Table 2) were significantly different (standardized residuals ≥|1.96|) from expectations. In other words, some directions of effects were more prominent in the sample than other directions of effects. Two findings merit attention. First, more families than expected showed reciprocal effects between adolescent positive affect and parental warmth and autonomy support. Second, daily associations with parental control were often parent-driven: more families than expected showed parent-driven effects between psychological control and positive affect, and between behavioral control and negative affect.

Moreover, heterogeneity in directionality was also found within families, such that the direction of effects depended on the specific parenting-affect association. As shown in Table 3, no single family consistently demonstrated only reciprocal effects, parent-driven effects, adolescent-driven effects, or no effects. When examining homogeneity across the models for either adolescent positive or negative affect, a few families (0% to 11.1%) demonstrated a similar direction of effects across associations. Thus, the direction of effects often did not generalize across the eight different parenting-affect associations for a given family.

**Table 3:** Direction of lagged parenting-affect effects summarized per family.

Direction of effects	Associations with PA & NA	Associations with PA	Associations with NA
	*N* (%)	*N* (%)	*N* (%)
1. Completely reciprocal	0 (0.0%)	6 (3.9%)	1 (0.7%)
2. Completely parent-driven	0 (0.0%)	2 (1.3%)	0 (0%)
3. Completely adolescent-driven	0 (0.0%)	1 (0.7%)	1 (0.7%)
4. No lagged effects at all	0 (0.0%)	3 (2.0%)	17 (11.1%)
5. Mix of lagged and no effects	153 (100.0%)	141 (89.8%)	135 (88.2%)
Total	153 (100.0%)	153 (100.0%)	153 (100.0%)

PA, positive affect; NA, negative affect.

#### Sensitivity analyses

We conducted several sensitivity analyses to investigate the robustness of the findings regarding heterogeneity in the direction of effects (H1). First, we conducted pre-registered analyses excluding participants with fewer than 50 observations (see Supplementary Table S5) and excluding outliers (see Supplementary Table S6). The results of both sensitivity analyses were in line with the main hypothesis. Again, more families than expected showed reciprocal effects between adolescent positive affect and parental autonomy support and warmth, although the proportion of families varied slightly. Second, because the two items of the behavioral control scale correlated weakly (*r* = .11), we ran separate models for each item, with the results shown in Supplementary Table S7. Again, more families than expected by chance did not have lagged effects between the separate behavioral control items and adolescent affect. There was, however, one interesting finding: more families than expected showed parent-driven effects with adolescent negative affect and Item 2 (strictness), but not with Item 1 (monitoring).

### Explaining heterogeneity in the direction of effects

Theoretical work suggests that parenting effects depend on the characteristics of the child^4,31^. Hence, to explore whether heterogeneity in directionality can be explained by adolescent attributes, we examined via non-preregistered analyses whether demographic characteristics (i.e., sex, education, and age) and two theoretically relevant personality traits (according to environmental sensitivity theories^7,8^) were related to the magnitude of the *absolute* family-specific effect sizes. No clear correlations were found with adolescent sex (0/16 significant) and educational level (2/16 significant; see Table 4). Correlations with adolescent age indicated that fluctuations in negative affect predicted both next-day psychological and behavioral control more strongly in younger (versus older) adolescents. Regarding personality traits, environmental sensitivity (4/16 significant) and neuroticism (7/16 significant) were correlated with several family-specific effects. Overall, these correlations suggest that adolescents with relatively higher environmental sensitivity had positive affect that was more affected by parenting, and that adolescents with relatively higher neuroticism had stronger daily linkages between perceived parenting and negative affect. Information about the measurements of environmental sensitivity and neuroticism can be found in the Supplementary Information.

**Table 4:** Moderators to explain heterogeneity in absolute effect sizes.

Family-specific lagged effect	Mean differences			Correlations	
	Sex (*t*-test)	Education (ANOVA)	Age	Environmental sensitivity^c^	Neuroticism
*Psychological control (PC)*					
PC → PA	− .14	.06*^a^	.04	.25**	.17*
PA → PC	− .22	.01	− .09	.08	.03
PC → NA	− .04	.00	− .07	− .01	.19*
NA → PC	.02	.00	− .18*	− .03	.13
*Behavioral control (BC)*					
BC → PA	− .10	.06	.13	.16*	.13
PA → BC	− .23	.03	− .14	.08	.11
BC → NA	− .12	.01	.08	.11	.28***
NA → BC	− .29	.01	− .18*	.11	.21**
*Autonomy support (AS)*					
AS → PA	− .29	.03	.02	.14	.22**
PA → AS	.13	.01	− .08	− .18*	− .08
AS → NA	− .31	.02	− .05	.13	.18*
NA → AS	.22	.01	− .14	− .12	.06
*Warmth (WA)*					
WA → PA	− .15	.03	− .04	.16*	− .03
PA → WA	− .06	.02	− .09	.00	− .06
WA → NA	− .04	.04*^b^	.00	.02	.05
NA → WA	− .24	.00	.01	.13	.29***

Cohen’s *d* is reported for sex (1 = male, 2 = female). Eta squared is reported for education (1 = low, 2 = moderate, 3 = high). PA = adolescent positive affect. NA = adolescent negative affect.

^a^Adolescents with a moderate education level showed stronger effects than those with high education levels.

^b^Adolescents with a low education level showed stronger effects than those with high education levels.

^c^Correlations between self-reported environmental sensitivity and the initial family-specific effect sizes are shown in Supplementary Figures S2-S9.

### Heterogeneity in the nature of reciprocal effects

The results of our exploratory analyses show that the sign (i.e., positive or negative) of the effects varied across families (for sample distributions of the family-specific effects, see Supplementary Table S3 and Supplementary Fig. S1). Both inhibiting and reinforcing reciprocal cycles were observed across families. However, inhibiting cycles were generally found in fewer families (than would be expected by chance), whereas reinforcing cycles were more prominent in the sample (than would be expected by chance; see Supplementary Table S4).

**Figure 4:**
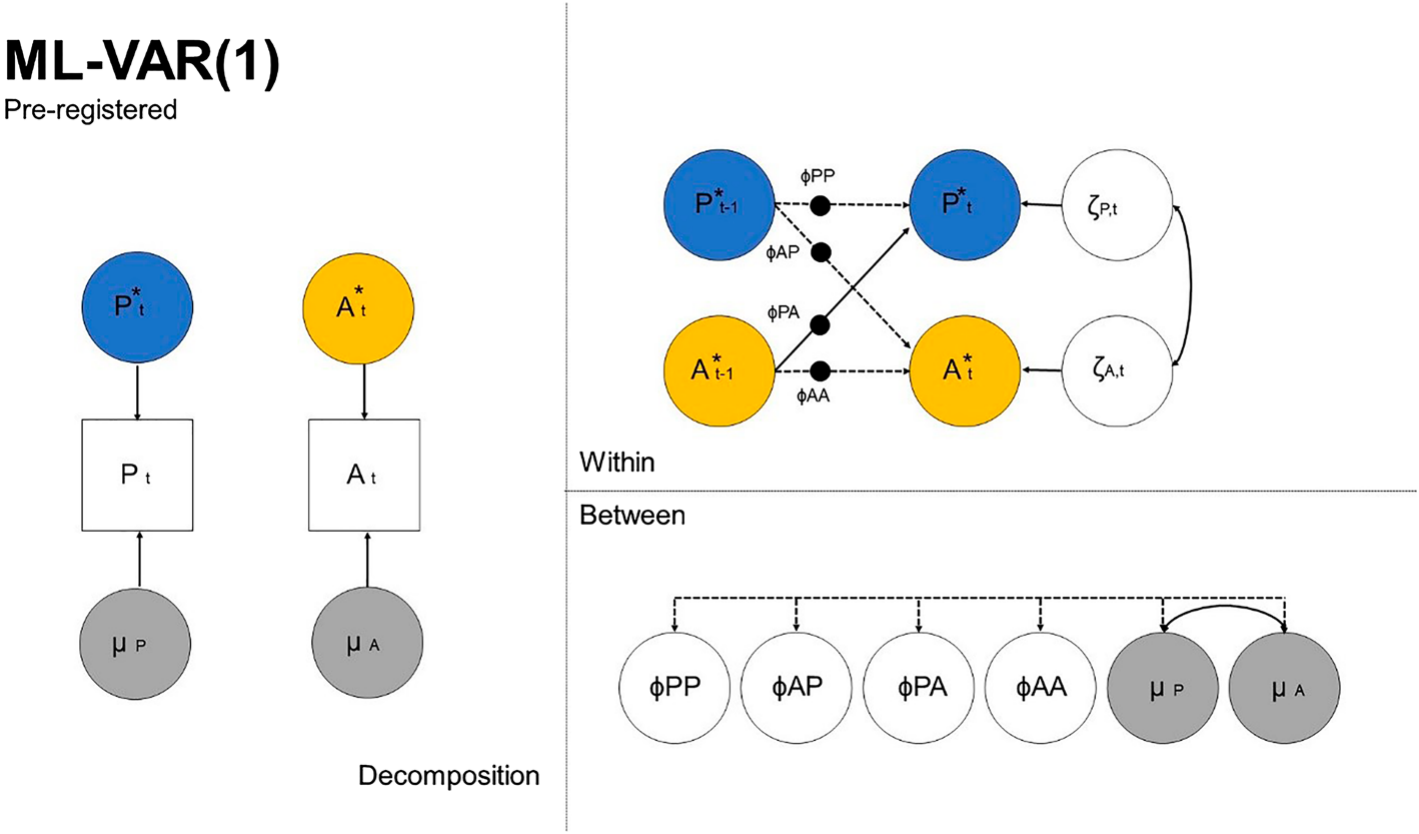
Specification of dynamic structural equation model. P = Parenting. A = Adolescent affect. Left: Variables are decomposed into a between-family (μ = family-specific mean) and within-family part (P*t and A*t = time-specific score of parenting and adolescent affect, respectively). Top right: Estimates at the within-family level, including the random (family-specific) cross-lagged (ϕAP and ϕPA), and autoregressive effects (ϕPP and AA) and the correlation between the innovations (ζ). Bottom right: Between-family level correlations between the random effects and means.

We illustrate the heterogeneity in reciprocal effects with the association between parental psychological control and adolescent positive affect. As depicted in Fig. 4, 20 families (12.9% of the sample) demonstrated a *negative* reinforcing cycle, meaning that both the parent- and adolescent-driven effects were negative in sign. In other words, in these families, increases in psychological control predicted decreases in positive affect, which in turn predicted an increase in psychological control. A *positive* reinforcing cycle was present in 10 families (6.5%); thus, in these families, psychological control predicted increases in next-day positive affect, and vice versa. A small number of families showed inhibiting cycles: in 2.6%, psychological control predicted decreases in positive affect, which predicted increases in psychological control; in 1.3%, psychological control predicted increases in positive affect, which predicted decreases in psychological control.

## Discussion

An enduring inquiry in developmental science concerns whether parents act in response to the well-being of their adolescent child, or whether adolescent well-being is the direct result of parenting practices^1,2^. Although reciprocity in parenting and adolescent well-being is now widely-accepted^2–4^, questions persist about the extent to which reciprocity findings—based on group-level or average patterns—accurately reflect individual-level parent-adolescent dynamics^14,20^. Therefore, in the current study, we examined whether different dyads demonstrate effects with different directions (i.e., reciprocal, parent-driven, adolescent-driven, or no effects; see Fig. 1)^5^. To do so, we adopted a novel idiographic approach and investigated 159 Dutch families’ unique 100-day dynamics between perceived parenting and adolescent affective well-being.

In line with our pre-registered hypothesis, different families (i.e., parent-adolescent dyads) demonstrated different directions of effects between perceived parenting (i.e., psychological control, behavioral control, autonomy support, and warmth) and adolescent affective well-being (both positive and negative) in everyday life. Whereas some families showed reciprocal day-to-day effects between dimensions of parenting and affective well-being, others showed only a parent-driven or an adolescent-driven effect, or no effects at all (for example, see Fig. 3). Importantly, even within the same family, the direction of effects did not generalize across associations among parenting and affective well-being dimensions. For instance, a family could demonstrate reciprocal effects between parental warmth and adolescent positive affect but a parent-driven effect between parental behavioral control and adolescent positive affect. Thus, although many developmental^4,31^ and parenting theories^3^ propose that influences between parents and adolescents are reciprocal, our findings suggest that this conclusion may only hold for a subgroup of families, and for certain sets of behaviors and emotions. In other words, every family has their own unique way of interacting in everyday life. This means that there may be potential drawbacks of a “one-size-fits-all” approach to family interventions, and that it may be important to move toward tailoring interventions to the specific dynamics and needs of a family^32,33^.

We found several meaningful adolescent attributes that explained the heterogeneity in directionality, and thus, speak to why parents and adolescents might differentially influence each other across families. First, heightened negative affect seemed to exhibit a stronger influence on parents’ controlling behaviors among younger adolescents. As parents generally exert less control as adolescents become older^34^, they might be more inclined to give older adolescents more space to deal with negative emotions than younger adolescents. Second, the positive affect of adolescents who reported higher trait levels of environmental sensitivity, specifically sensory processing sensitivity (SPS)^9^, seemed more strongly influenced by parenting behaviors. This finding is consistent with environmental sensitivity theories, which propose that an underlying phenotypic trait, such as SPS, leads to a higher responsivity to the environment^6,35^. Third, daily fluctuations in parenting were more strongly tied to daily fluctuations in negative affect among adolescents with higher neuroticism, converging with prior work indicating that neuroticism is associated with enhanced negative feelings, especially in reaction (negative) events in daily life^36^.

In addition to the direction of effects, our findings reveal insights into the nature of everyday parent-adolescent dynamics. Overall, we found that reinforcing cycles (e.g., more warmth → more positive affect → more warmth) are more prominent than inhibiting cycles (e.g., more warmth → more positive affect → less warmth). From a dynamic systems perspective, reciprocal influences can result in either change or growth by reinforcing feedback loops or stabilization by inhibiting feedback loops^13^. This is consistent with our findings highlighting the prominence of these reinforcing reciprocal cycles during periods of change (i.e., adolescence). Indeed, adolescence is a period in which parent–child relationships need to be realigned^37^. A second COVID-19 lockdown, however, started halfway through the study^38^, which could have destabilized the family system. Examining non-linear dynamics, preferably while linking short-term dynamics to longer-term development, is a promising avenue for future work, as it may help unravel how everyday family dynamics become a driving force in developmental trajectories.

This pre-registered idiographic study examined day-to-day parent-adolescent dynamics at the individual family level by rigorously analyzing more than 14,000 daily diaries of 159 adolescents. Despite these strengths, our findings must be considered in light of several limitations. First, different inference criteria could have revealed different effects; this is important to consider in emerging idiographic research, as strong precedents for such criteria are lacking. Here family-specific inferences were based on the smallest effect size of interest (SESOI; β ≥ .05) rather than significance levels^29,39^. Preferably, future intensive longitudinal studies with more data points per family will combine a SESOI with a threshold of statistical significance^40^. Second, we studied adolescent-perceived parenting, and prior research has shown discrepancies between parents and adolescents in their perception of daily parenting behavior^41^. Future work is needed to explore the heterogeneity in how parents perceive daily parent-adolescent dynamics. Third, the current day-to-day findings might not generalize across timescales, so other timescales also warrant attention in future studies, such as a momentary (instead of a daily) timescale^27^. Fourth, the sample consisted of more female than male adolescents, and the majority were highly educated, which might have limited our ability to detect sex and educational differences. Future studies with larger and more diverse samples are needed to gain more insight into individual factors that might explain heterogeneity among adolescents.

In conclusion, most contemporary parenting theories posit that parents and children mutually affect one another^2,4^, especially during adolescence, when children become more active agents within the parent–child relationship^3^. Our findings, however, point towards a more nuanced understanding that was achieved by adopting a novel idiographic approach to the investigation of families’ unique daily dynamics: The direction of day-to-day influences between parenting and adolescent well-being depends on the family and on the parenting behaviors and adolescent emotions under consideration. Environmental sensitivity and neuroticism appear to be promising traits for understanding why some adolescents are more strongly affected by parenting than are others. Hence, rather than being a homogeneous phenomenon, the ways in which parents and adolescents influence each other in everyday life is unique to each family. Moving towards an idiographic parenting science, with a focus on individual families, is needed to unravel the complex reality of parenting adolescents. This, in turn, may ultimately inform interventions tailored to the unique dynamics and needs of unique families.

## Method

### Participants

A total of 159 adolescent-parent dyads participated in the “100 days of my life” study (https://osf.io/5mhgk/). The adolescents were between 12 and 16 years old (*M*_age_ = 13.1, *SD*_age_ = 1.22), and 62% were female (36% male, 2% neither female nor male). Most were born in the Netherlands (89%), and some in other European countries (6%), or counties in Asia (2%), North America (1%), South America (1%), or Africa (1%). Moreover, 15% of the adolescents followed pre-vocational secondary education or vocational training, 30% higher general secondary education, 51% pre-university secondary education, and 5% a mixed educational track.

Adolescents reported on one participating primary caregiver of choice: biological mothers (79%), fathers (19%), or other caregivers (*n* = 1 adoption mother, *n* = 1 s mother, *n* = 1 stepfather)—hereafter called parents. Parents were on average 45.3 years old (*SD* = 4.54, Range = 33–55), and most were born in the Netherlands (87%). Some were born in other European countries (6%), Asia (3%), North America (1%), South America (1%), Africa (1%), and Australia (1%). Ten percent of the parents completed up to high school, 25% completed vocational/technical training, 62% graduated from college or university, and 3% gave insufficient information to determine their educational level.

### Procedure

Most parent-adolescent dyads were recruited via two high schools in the Netherlands, which offered low to high secondary educational tracks to 1,300 and 2,000 students, respectively. Families were informed about the study through class visits, email, and posters. Other families were informed through personal communication, social media, and a newsletter to participants of a former project. Interested families received a detailed briefing via a video call, after which both parents and adolescents signed an online informed consent form. Parents also provided informed consent for the participation of their underage adolescent. One dyad (i.e., composed of an adolescent between 12 and 16 years old and one parent with whom they had daily contact) could participate per family. Both members of the dyad needed to own a smartphone in order to participate.

For 100 consecutive days (Oct 26, 2020 until Feb 2, 2021), adolescents and parents answered one daily questionnaire via the Ethica Data app, which they installed on their own smartphone. The questionnaires took approximately 3 to 5 min to complete. Participants were prompted in the evening between 7 and 10PM, depending on their preference. They received a maximum of four automatic reminders in the evening and one final call at 7AM the next morning. Most of the daily diaries (86%) were completed before this final call.

To ensure high compliance, we added several motivational features. First, participants received a monetary reward for each completed questionnaire and bonuses if they completed 10 questionnaires in a row and 100 questionnaires in total. Adolescents could receive up to €100 (≈US$ 121). Second, every day, €10 was raffled off to two adolescents who completed the daily questionnaire. Third, participants could compensate for missing questionnaires by extending their participation by another 25 days, which led to an average participation length of 107 days.

During their 107 days of participation, adolescents completed an average of 87% of the prompted diaries, resulting in 93 completed diaries per adolescent (range 24–108). All available data, including incomplete diaries, were used. The total number of observations per variable ranged from 14,512 to 14,819. This study was approved by the Ethical Committee of Tilburg University (RP250), and all methods were performed in accordance with the relevant guidelines and regulations. More detailed information about the procedure can be found online: https://osf.io/5mhgk/.

### Measures

All items were scored on a visual analogue scale (VAS) ranging from 0 (*Not at all*) to 100 (*Very much*).

#### Parental psychological control

Parental psychological control involves regulating others’ thoughts and emotions through manipulative behaviors, including (a) constraining verbal expression, (b) guilt induction, and (c) love withdrawal^42^. To measure these parenting behaviors, adolescents rated three items that were adapted from an existing 4-item daily diary scale^43^. The items were: “When I wanted to say something, my parent started to talk about something else” (constraining verbal expressions), “My parent blamed me for the problems at home” (guilt induction), and “My parent was less affectionate towards me when I did not see things his/her way” (love withdrawal). Multilevel confirmatory factor analysis indicated moderate internal consistency at the within-family level (ω = .61) and excellent internal consistency at the between-family level (ω = .83)^44^.

#### Parental behavioral control

Parental behavioral control involves regulating others’ behavior through (a) rules, regulations, and restrictions and (b) actively monitoring whereabouts and activities^45^. To capture both facets, adolescents rated two items, which were adapted from prior work^46,47^. The items were “My parent was strict” (rule setting) and “I had to tell my parent what I did, with whom and where” (monitoring). Internal consistency, measured with the inter-item correlation, was insufficient at the within-family level (*r* = .11, *p* < .001) and good at the between-family level (*r* = .50, *p* < .001). Hence, although the two items co-fluctuated to some extent, the items likely reflected different parenting practices. We report the pre-registered analyses of the subscale in the main text and then examined differences using each item separately in sensitivity analyses (see Supplementary Table S7).

#### Parental autonomy support

Parental autonomy support is defined by (a) the provision of choice and allowance of independent decision-making and (b) acknowledgment and interest in the adolescent’s perspective^48^. To capture both components, adolescents rated two items adapted from a 4-item daily autonomy support scale^43^. The items were: “My parent allowed me to make my own plans” (independent decision-making) and “My parent took my point of view into account” (acknowledgment of perspective). Internal consistency of the 2-item scale was sufficient at the within-family (*r* = .46, *p* < .001) and good at the between-family level (*r* = .76* p* < .001).

#### Parental warmth

Parental warmth includes (a) provision of affection and (b) parental care and responsiveness^48^, which were rated by adolescents with two items. The items were adapted from a Dutch daily diary study^49^. The items were: “The relationship with my parent was enjoyable” and “My parent showed me that she/he cares for me.” Internal consistency of the two items was good at both the within-family (*r* = .64, *p* < .001) and between-family levels (*r* = .85,* p* < .001).

#### Adolescent affective well-being

Affective well-being can be defined by high levels of positive affect (i.e., pleasant, desirable feelings) and low levels of negative affect (i.e., unpleasant, undesirable feelings)^50^. Therefore, to measure daily affective well-being, we used five items from the Positive and Negative Affect Schedule for Children (PANAS-C)^51^. These items were chosen based on the psychometric properties of the Dutch scale in an adolescent sample from previous work^30^. Positive affect was measured with two items (“joyful” and “happy”), and negative affect with three items (“mad”, “afraid”, and “sad”). Internal consistency of the positive affect scale was good at both the within-family (*r* = .76,* p* < .001) and between-family level (*r* = .95,* p* < .001). The internal consistency of the negative affect scale was good at the within-family level (ω = .71) and excellent at the between-family level (ω = .92).

### Preregistered analytical approach

To assess how perceived parenting and adolescent affect predicted each other in each family, we used dynamic structural equation modelling (DSEM)^52^ in M*plus* 8.5. This relatively novel analytical technique combines the strengths of structural equation modeling, multilevel modeling, and *N* = 1 time series analyses—and can yield both insights into within-family effects at the group level (i.e., averaged effects) as well as at the level of the individual family (i.e., family-specific effects). Preliminary analyses confirmed that the data met the assumption of weak stationarity because time (i.e., days in the study) explained little-to-no variance (0.0–0.1%) in the parenting and affect variables. Eight lag-1 multilevel vector autoregressive (ML-VAR(1)) models (see Fig. 4) were estimated: 4 (parenting dimensions) × 2 (affect dimensions). The within-family bi-variate cross-lagged effects were specified as random effects to estimate these (family-specific) effects for each individual family separately. The within-person coupling reliability (WPCR) of the family-specific parenting-affect couplings ranged between .72 and .97 for the couplings concerning positive affect, and between .45 and .75 for couplings concerning negative affect^53^. To account for unequal time intervals between measurements due to missing data, the option TINTERVAL was set to 1 (i.e., 1 day). All data points were placed in this equal day-to-day time interval and missing data were inserted into time intervals without data. Due to the Kalman filter implemented in DSEM, all available observations were used in the DSEM analyses^52,54^.

Model convergence was inspected using two criteria: (1) PSR lower than 1.1 (potential scale reduction factor) and (2) whether the trace plots of the parameters look like fat caterpillars, especially the plots of the cross-lagged parameters^54^. We used 40,000 iterations and a thinning factor of 10 in our final models. If the models did not converge with all random effects, fixed autoregressive effects were estimated.

#### Inference criteria

We extracted the family-specific standardized cross-lagged effects (i.e., STDYX standardization) from the ML-VAR(1) models by using the R package “M*plus* Automation”^55^. As pre-registered (https://osf.io/7n2jx/), these standardized effects were interpreted based on the smallest effect size of interest (SESOI) of .05^29,40^. A standardized within-family cross-lagged effect of .05 can be considered a small(-to-moderate) effect according to recent guidelines^56^. Hence, we interpreted standardized family-specific cross-lagged effects smaller than .05 as null effects (− .05 > β < .05), effects with a size of β ≥ .05 as positive effects, and effects with a size of β ≤ − .05 as negative effects.

### Additional (non-preregistered) analyses

We additionally explored whether the proportions of families showing reciprocal, parent-driven, adolescent-driven, and null effects were different from what would be expected by chance (i.e., 25% of families showing each type of effect). To do so, we used chi-square tests, reviewing standardized residuals ( ≥|1.96|) to interpret which effects were significantly more or less prominent in our sample. Moreover, we tested whether demographic factors and the two personality traits could explain differences in terms of (absolute) effect sizes. Specifically, we tested for sex differences (*t*-test), differences between adolescents with varying educational levels (ANOVA), and correlations with age and trait levels of environmental sensitivity and neuroticism.

### Supplementary Information

Supplementary Information.

## Data Availability

The preregistered hypothesis and analytical approach, code, output, and data (https://osf.io/7n2jx), and codebook of the data collection (https://osf.io/5mhgk) are shared on the Open Science Framework.
