# Supplementary material for: The direction of effects between parenting and adolescent affective well-being in everyday life is family specific
Source: Sci Rep. 2023 Sep 26;13:16106. doi: 10.1038/s41598-023-43294-5 (PMC10522680; doi:10.1038/s41598-023-43294-5)

**Supplemental Online Material**

**DSEM Analyses**

**Table S1**

|  | **Models with positive affect** | | | | | | | | | | | | | | |
| --- | --- | --- | --- | --- | --- | --- | --- | --- | --- | --- | --- | --- | --- | --- | --- |
|  | 1. Psychological control | | |  | 1. Behavioral control | | |  | 1. Autonomy support | | |  | 1. Warmth | | |
| Fixed lagged effects  (within-family average) | Est. | Est.  St. | 95% CI |  | Est. | Est. St. | 95% CI |  | Est. | Est.  St. | 95% CI |  | Est. | Est.  St. | 95% CI |
| Parenting 🡪 Parenting | **0.32** | **.32** | **[0.28, 0.36]** |  | **0.31** | **.31** | **[.27, .34]** |  | **0.31** | **.31** | **[.27, .34]** |  | **0.28** | **.28** | **[0.24, 0.32]** |
| Affect 🡪 Affect | **0.26** | **.26** | **[0.21, 0.30]** |  | **0.35** | **.35** | **[.31, .39]** |  | **0.27** | **.27** | **[.23, .30]** |  | **0.29** | **.29** | **[0.25, 0.33]** |
| Parenting **🡪**  Affect | -0.03 | -.02 | [-0.12, 0.06] |  | 0.00 | -.01 | [-.04, .03] |  | **0.03** | **.05** | **[.01, .06]** |  | **0.09** | **.09** | **[0.05, 0.13]** |
| Affect 🡪 Parenting | -0.01 | -.03 | [-0.03. 0.01] |  | 0.02 | .02 | [-.01, .05] |  | **0.08** | **.07** | **[.04, .12]** |  | **0.06** | **.07** | **[0.04, 0.09]** |
| Random effects  (between-family variance) | σ^2^ | Ratio:  *SD*/Est | 95% CI |  | σ^2^ | Ratio:  *SD*/Est | 95% CI |  | σ^2^ | Ratio:  *SD*/Est | 95% CI |  | σ^2^ | Ratio:  *SD*/Est | 95% CI |
| Parenting 🡪 Parenting | 0.04 | 0.63 | [0.03, 0.06] |  | 0.03 | 0.60 | [0.02, 0.05] |  | 0.04 | 0.65 | [.03, .05] |  | 0.03 | 0.63 | [0.02,0.04] |
| Affect 🡪 Affect | 0.04 | 0.77 | [0.03, 0.06] |  | 0.04 | 0.60 | [0.03, 0.06] |  | 0.03 | 0.64 | [.02, .04] |  | 0.03 | 0.63 | [0.02, 0.05] |
| Parenting **🡪**  Affect | 0.14 | 12.07 | [0.08, 0.23] |  | 0.03 | 55.78 | [0.02, 0.04] |  | 0.01 | 3.33 | [.01, .02] |  | 0.03 | 1.86 | [0.02, 0.04] |
| Affect 🡪 Parenting | 0.01 | 6.45 | [0.00, 0.01] |  | 0.01 | 6.00 | [0.01, 0.02] |  | 0.03 | 2.17 | [.02, .05] |  | 0.01 | 1.91 | [0.01, 0.02] |

*DSEM analyses with Perceived Parenting and Adolescent Positive Affect*

*Note*. All data are adolescent-reported. Bold effects are significant fixed effects. Est = unstandardized estimate. Est. St. = standardized estimate (i.e., STDYX standardization). *p* = one-sided *p*-value. 95% CI = Bayesian Credible Intervals. Ratio: *SD*/Est = standard deviation fixed effect ratio, to inspect whether variance is meaningful with a criterium of ≥ 0.25 (Bolger et al., 2019).

**Table S2**

|  | **Models with negative affect** | | | | | | | | | | | | | | |
| --- | --- | --- | --- | --- | --- | --- | --- | --- | --- | --- | --- | --- | --- | --- | --- |
|  | 1. Psychological control | | |  | 1. Behavioral control | | |  | 1. Autonomy support | | |  | 1. Warmth | | |
| Fixed lagged effects  (within-family average) | Est. | Est.  St. | 95% CI |  | Est. | Est.  St. | 95% CI |  | Est. | Est.  St. | 95% CI |  | Est. | Est.  St. | 95% CI |
| Parenting 🡪 Parenting | **0.27** | **.27** | **[0.25, 0.29]** |  | **0.24** | **.24** | **[0.21, 0.28]** |  | **0.25** | **.25** | **[0.22, 0.29]** |  | **0.25** | **.25** | **[0.21, 0.29]** |
| Affect 🡪 Affect | **0.32** | **.32** | **[0.31, 0.34]** |  | **0.36** | **.36** | **[0.32, 0.40]** |  | **0.28** | **.28** | **[0.25, 0.32]** |  | **0.30** | **.30** | **[0.26, 0.34]** |
| Parenting **🡪**  Affect | **0.05** | **.04** | **[0.02, 0.09]** |  | **0.04** | **.05** | **[0.02, 0.07]** |  | -0.01 | -.01 | [-0.02, 0.01] |  | -0.02 | -.03 | [-0.04, 0.00] |
| Affect 🡪 Parenting | 0.00 | .00 | [-0.03. 0.02] |  | -0.02 | -.01 | [-0.05, 0.01] |  | 0.00 | .00 | [-0.04, 0.04] |  | -0.02 | -.02 | [-0.05, 0.02] |
| Random effects  (between-family variance) | σ^2^ | Ratio:  *SD*/Est | 95% CI |  | σ^2^ | Ratio:  *SD*/Est | 95% CI |  | σ^2^ | Ratio:  *SD*/Est | 95% CI |  | σ^2^ | Ratio:  *SD*/Est | 95% CI |
| Parenting 🡪 Parenting | - | - | - |  | 0.03 | 0.72 | [0.02, 0.04] |  | 0.03 | 0.69 | [0.02, 0.04] |  | 0.03 | 0.69 | [0.02, 0.04] |
| Affect 🡪 Affect | - | - | - |  | 0.04 | 0.56 | [0.03, 0.06] |  | 0.03 | 0.62 | [0.02, 0.04] |  | 0.03 | 0.60 | [0.02, 0.05] |
| Parenting **🡪**  Affect | 0.01 | 2.15 | [0.01, 0.03] |  | 0.01 | 2.50 | [0.01, 0.02] |  | 0.00 | 7.82 | [0.00, 0.01] |  | 0.01 | 3.37 | [0.00, 0.01] |
| Affect 🡪 Parenting | 0.02 | 40.82 | [0.01, 0.02] |  | 0.01 | 6.71 | [0.01, 0.02] |  | 0.02 | 47.14 | [0.01, 0.03] |  | 0.02 | 9.43 | [0.01, 0.03] |

*DSEM analyses with Perceived Parenting and Adolescent Negative Affect*

*Note*. All data are adolescent-reported. Bold effects are significant fixed effects. Est = unstandardized estimate. Est. St. = standardized estimate (i.e., STDYX standardization). *p* = one-sided *p*-value. 95% CI = Bayesian Credible Intervals. Ratio: *SD*/Est = standard deviation fixed effect ratio, to inspect whether variance is meaningful with a criterium of ≥ 0.25 (Bolger et al., 2019).

**Family-Specific Effect Sizes**

**Table S3**

*Sample Distribution of the Effect Sizes of the Family-Specific Cross-Lagged Effects*

|  | **Adolescent positive affect** | | |  |
| --- | --- | --- | --- | --- |
| Lagged effect | Negative effect | Null effect | Positive effect | Range of  effect sizes |
|  | *N* (%) | *N* (%) | *N* (%) |  |
| ***Psychological control*** |  |  |  |  |
| Parenting 🡪 Affect | 58 (37%) | 66 (43%) | 31 (20%) | -.63 to .48 |
| Affect 🡪 Parenting | 40 (26%) | **95 (61%)** | 20 (13%) | -.65 to .22 |
| ***Behavioral control*** |  |  |  |  |
| Parenting 🡪 Affect | 44 (28%) | 77 (49%) | 36 (23%) | -.40 to .48 |
| Affect 🡪 Parenting | 21 (13%) | **95 (61%)** | 41 (26%) | -.24 to .34 |
| ***Autonomy support*** |  |  |  |  |
| Parenting 🡪 Affect | 18 (11%) | 71 (45%) | 69 (44%) | -.16 to .40 |
| Affect 🡪 Parenting | 12 (8%) | 63 (40%) | **83 (53%)** | -.31 to .40 |
| ***Warmth*** |  |  |  |  |
| Parenting 🡪 Affect | 13 (8%) | 41 (26%) | **105 (66%)** | -.18 to .58 |
| Affect 🡪 Parenting | 15 (9%) | 56 (35%) | **88 (55%)** | -.15 to .35 |
|  | **Adolescent negative affect** | | |  |
| Lagged effect | Negative effect | Null effect | Positive effect | Range of  effect sizes |
|  | *N* (%) | *N* (%) | *N* (%) |  |
| ***Psychological control*** |  |  |  |  |
| Parenting 🡪 Affect | 4 (3%) | **112 (72%)** | 39 (25%) | -.12 to .15 |
| Affect 🡪 Parenting | 26 (17%) | **96 (62%)** | 33 (21%) | -.67 to .35 |
| ***Behavioral control*** |  |  |  |  |
| Parenting 🡪 Affect | 16 (10%) | 61 (39%) | **80 (51%)** | -.17 to .50 |
| Affect 🡪 Parenting | 31 (20%) | **112 (71%)** | 14 (9%) | -.24 to .20 |
| ***Autonomy support*** |  |  |  |  |
| Parenting 🡪 Affect | 28 (18%) | **116 (73%)** | 14 (9%) | -.22 to .10 |
| Affect 🡪 Parenting | 21 (13%) | **119 (75%)** | 18 (11%) | -.17 to .14 |
| ***Warmth*** |  |  |  |  |
| Parenting 🡪 Affect | 34 (21%) | **117 (74%)** | 8 (5%) | -.22 to .10 |
| Affect 🡪 Parenting | 40 (25%) | **87 (55%)** | 32 (20%) | -.17 to .14 |

*Note*. Negative effect is = β ≤ -.05. Null effect = -.05 < β < .05. Positive effect = β ≥ .05.

Group size in bold is a majority in the given association.

**Table S4**

*Number of Families with Inhibiting or Reinforcing Reciprocal Effects*

| Reciprocal cross-lagged effect | Reciprocal effects | | | |  |
| --- | --- | --- | --- | --- | --- |
|  | Inhibiting cycle | | Reinforcing cycle | |  |
|  | (1)  + - | (2)  - + | (3)  - - | (4)  + + |  |
|  | *N* (%) | *N* (%) | *N* (%) | *N* (%) | Total *N* (100%) |
| ***Positive affect*** |  |  |  |  |  |
| 1. Psychological control - PA | 4 (2.6%) | 2 (1.3%)^↓^ | 20 (12.9%)^↑^ | 10 (6.5%) | 155 |
| 3. Behavioral control - PA | 2 (1.3%)^↓^ | 2 (1.3%)^↓^ | 12 (7.6%) | 20 (12.7%)^↑^ | 157 |
| 5. Autonomy support - PA | 2 (1.3%)^↓^ | 4 (2.5%)^↓^ | 6 (3.8%)^↓^ | 46 (29.1%)^↑^ | 158 |
| 7. Warmth - PA | 3 (1.9%)^↓^ | 0 (0.0%)^↓^ | 4 (2.5%)^↓^ | **80 (50.3%)**^↑^ | 159 |
| ***Negative affect*** |  |  |  |  |  |
| 2. Psychological control - NA | 7 (4.5%) | 1 (0.6%)^↓^ | 2 (1.3%)^↓^ | 18 (11.6%)^↑^ | 155 |
| 4. Behavioral control - NA | 3 (1.9%) | 0 (0.0%)^↓^ | 13 (8.3%)^↑^ | 12 (7.6%)^↑^ | 157 |
| 6. Autonomy support - NA | 1 (0.6%) | 2 (1.3%) | 10 (6.3%)^↑^ | 5 (3.2%) | 158 |
| 8. Warmth - NA | 0 (0.0%)^↓^ | 2 (1.3%)^↓^ | 21 (13.2%)^↑^ | 6 (3.8%) | 159 |

*Note*. “+ -” = positive parent-driven effect and negative adolescent-driven effect, “- +” = negative parent-driven effect and positive adolescent-driven effect. “- -” = negative parent- and adolescent-driven effect. “+ +” = positive parent- and adolescent-driven effect. PA = adolescent positive affect. NA = adolescent negative affect. Group size in bold is a majority in the given association.

^↑^ Proportion greater than would be expected by chance (i.e., 25%)

^↓^ Proportion less than would be expected by chance (i.e., 25%)

**Figure S1**

*Sample Distribution of Family-Specific Effect Sizes of Parental Psychological Control on Adolescent Positive Affect*


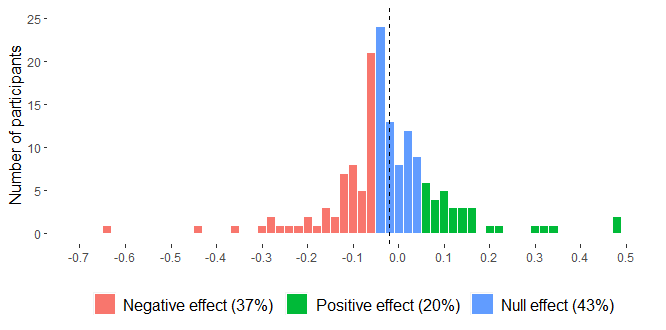


*Note*. The dotted line is the average effect in the sample (β = -.02, see Table S1). The sample distribution of all family-specific effect sizes is reported in Table S3.

**Measurement of Personality Traits**

Neuroticism was measured with a 6-item scale of the Big Five Inventory II (Denissen et al., 2020). The six items were answered using a response scale from 1 (*totally disagree*) to 5 (*totally agree*). The neuroticism scale showed good reliability, with a Cronbach’s alpha of .79.

Environmental sensitivity was measured using a 12-item version of the Hypersensitivity Child Scale (HSC; Pluess et al., 2018). The HSC consists of three subscales: Ease of Excitation (5 items, e.g. “I get nervous when I have to do a lot in little time”), Aesthetic Sensitivity (4 items, e.g. “I notice when small things have changed in my environment”), and Low Sensory Threshold (3 items, e.g. “I don’t like loud noises”) (Weyn et al., 2021). The items were rated on a scale from 1 (*not at all*) to 7 (*extremely*), and the scale showed good reliability, with a Cronbach’s Alpha of .74.

Both neuroticism and environmental sensitivity were reported by the adolescent at the start of the study (baseline). For more information see [*masked*].

**Sensitivity Analyses**

**Table S5**

*Excluding Participants (n = 7) with Less Than 50 Observations*

| Cross-lagged association | Direction of effects | | | | Total *N* |
| --- | --- | --- | --- | --- | --- |
|  | Reciprocal  *N* (%) | Parent-driven  *N* (%) | Adolescent-driven  *N* (%) | No effects  *N* (%) |  |
| ***Positive affect*** |  |  |  |  |  |
| 1. Psychological control | 25 (16.9%)^↓^ | 44 (29.7%) | 28 (18.9%) | 51 (34.5%)^↑^ | 148 |
| 2. Behavioral control | 36 (24.0%) | 35 (23.2%) | 26 (17.3%)^↓^ | 53 (35.3%)^↑^ | 150 |
| 3. Autonomy support | 56 (37.1%)^↑^ | 28 (18.5%) | 35 (23.2%) | 32 (21.2%) | 151 |
| 4. Warmth | **81 (53.3%)**^↑^ | 22 (14.5%)^↓^ | 18 (11.8%)^↓^ | 31 (20.4%) | 152 |
| ***Negative affect*** |  |  |  |  |  |
| 5. Psychological control | 26 (17.6%)^↓^ | 12 (8.1%)^↓^ | 33 (22.3%) | **77 (52.0%)**^↑^ | 148 |
| 6. Behavioral control | 29 (19.3%) | 58 (38.7%)^↑^ | 19 (12.7%)^↓^ | 44 (29.3%) | 150 |
| 7. Autonomy support | 18 (11.9%)^↓^ | 25 (16.6%)^↓^ | 22 (14.6%)^↓^ | **86 (57.0%)**^↑^ | 151 |
| 8. Warmth | 27 (17.8%)^↓^ | 10 (6.6%)^↓^ | 42 (27.6%) | 73 (48.0%)^↑^ | 152 |

*Note***.** Group size in bold is a majority in the given association.

^↑^ Proportion greater than would be expected by chance

^↓^ Proportion less than would be expected by chance

**Table S6**

*Excluding Outliers*

| Cross-lagged association | Direction of effects | | | | Total *N* |
| --- | --- | --- | --- | --- | --- |
|  | Reciprocal  *N* (%) | Parent-driven  *N* (%) | Adolescent-driven  *N* (%) | No effects  *N* (%) |  |
| ***Positive affect*** |  |  |  |  |  |
| 1. Psychological control | 37 (23.9%) | 57 (36.8%)^↑^ | 12 (7.7%)^↓^ | 49 (31.6%) | 155 |
| 2. Behavioral control | 31 (19.7%) | 23 (14.6%)^↓^ | 36 (22.9%) | 67 (42.7%)^↑^ | 157 |
| 3. Autonomy support | 71 (44.9%)^↑^ | 15 (9.5%)^↓^ | 45 (28.5%) | 27 (17.1%)^↓^ | 158 |
| 4. Warmth | **86 (54.1%)**^↑^ | 24 (15.1%)^↓^ | 29 (18.2%) | 20 (12.6%)^↓^ | 159 |
| ***Negative affect*** |  |  |  |  |  |
| 5. Psychological control | 22 (14.2%)^↓^ | 21 (13.5%)^↓^ | 23 (14.8%)^↓^ | **89 (57.4%)**^↑^ | 155 |
| 6. Behavioral control | 13 (8.3%)^↓^ | 29 (18.5%) | 6 (3.8%)^↓^ | **109 (69.4%)**^↑^ | 157 |
| 7. Autonomy support | 18 (11.4%)^↓^ | 21 (13.3%)^↓^ | 21 (13.3%)^↓^ | **98 (62.0%)**^↑^ | 158 |
| 8. Warmth | 20 (12.6%)^↓^ | 32 (20.1%) | 28 (17.6%)^↓^ | 79 (49.7%)^↑^ | 159 |

*Note***.** Group size in bold is a majority in the given association.

^↑^ Proportion greater than would be expected by chance

^↓^ Proportion less than would be expected by chance

**Table S7**

*Direction of Effects Analyzed Per Item of Behavioral Control*

| Cross-lagged association | Direction of effects | | | |  |
| --- | --- | --- | --- | --- | --- |
|  | Reciprocal  *N* (%) | Parent-driven  *N* (%) | Adolescent-driven  *N* (%) | No effects  *N* (%) | Total *N* |
| ***Positive affect*** |  |  |  |  |  |
| 1. Item strictness | 17 (11%)^↓^ | 45 (29%) | 13 (8%)^↓^ | **82 (52%)**^↑^ | 157 |
| 2. Item monitoring | 34 (22%) | 29 (19%) | 30 (20%) | 60 (39%)^↑^ | 153 |
| ***Negative affect*** |  |  |  |  |  |
| 3. Item strictness | 22 (14%)^↓^ | 32 (20%) | 29 (19%) | 74 (47%)^↑^ | 157 |
| 4. Item monitoring | 23 (15%)^↓^ | 61 (40%)^↑^ | 10 (7%)^↓^ | 59 (39%)^↑^ | 153 |

*Note***.** Group size in bold is a majority in the given association. Item strictness = “My parent was strict”. Item monitoring = “I had to tell my parent what I did, with whom, and where”.

^↑^ Proportion greater than would be expected by chance

^↓^ Proportion less than would be expected by chance

**Correlations Between the Family-Specific Effect Sizes and Environmental Sensitivity**

**Figure S2**

*Correlations of Environmental Sensitivity with the Family-Specific Day-to-Day Effects between Parental Psychological Control and Adolescent Positive Affect*

**
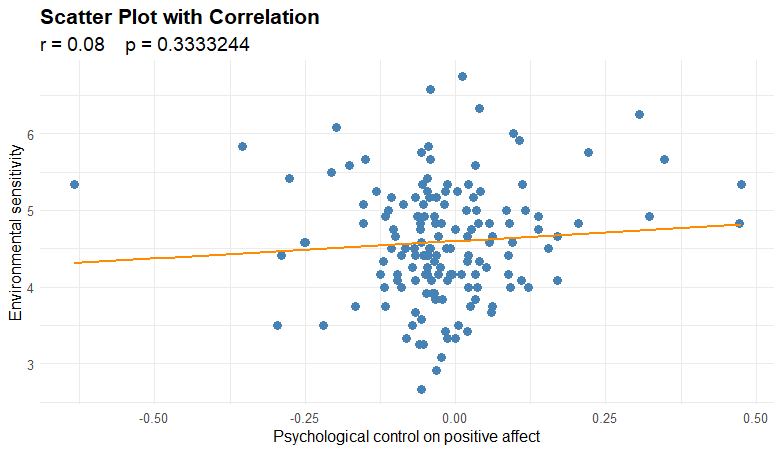

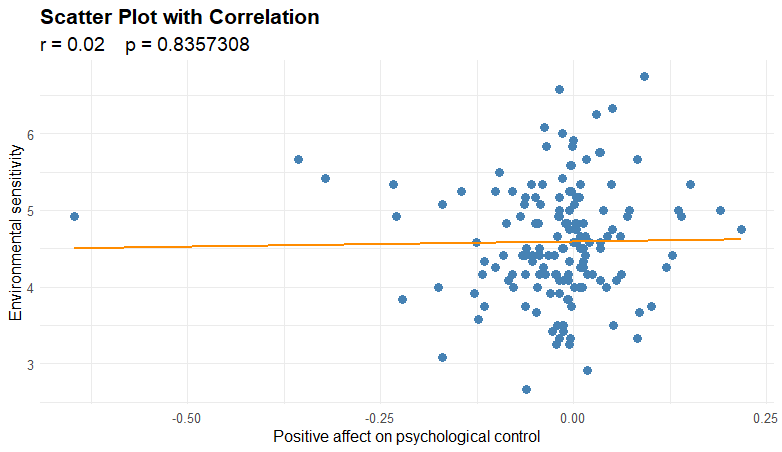
**

**Figure S3**

*Correlations of Environmental Sensitivity with the Family-Specific Day-to-Day Effects between Parental Psychological Control and Adolescent Negative Affect*

**
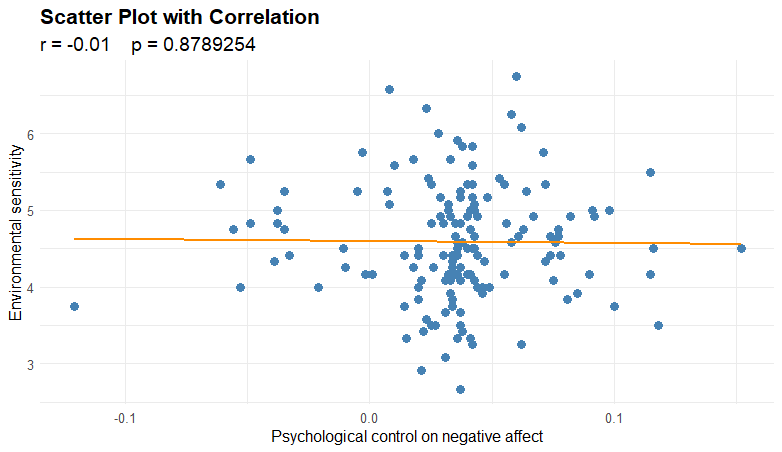
**

**
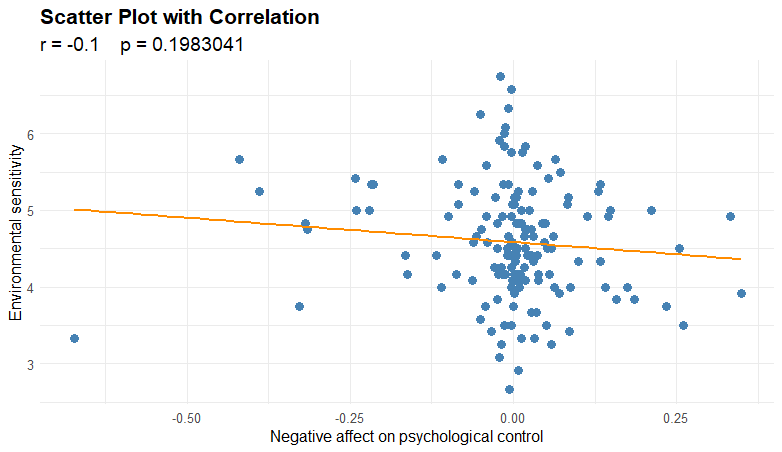
**

**Figure S4**

*Correlations of Environmental Sensitivity with the Family-Specific Day-to-Day Effects between Parental Behavioral Control and Adolescent Positive Affect*


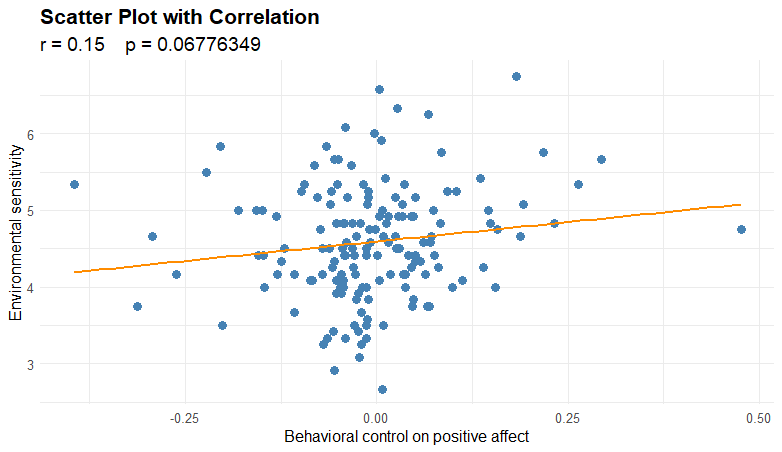


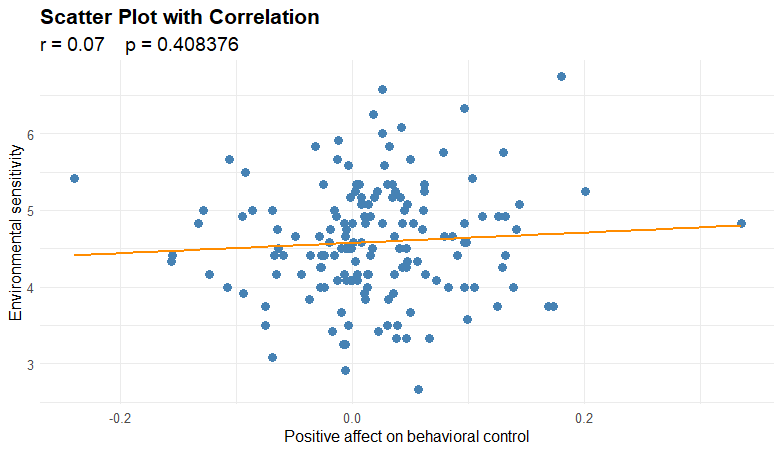


**Figure S5**

*Correlations of Environmental Sensitivity with the Family-Specific Day-to-Day Effects between Parental Behavioral Control and Adolescent Negative Affect*


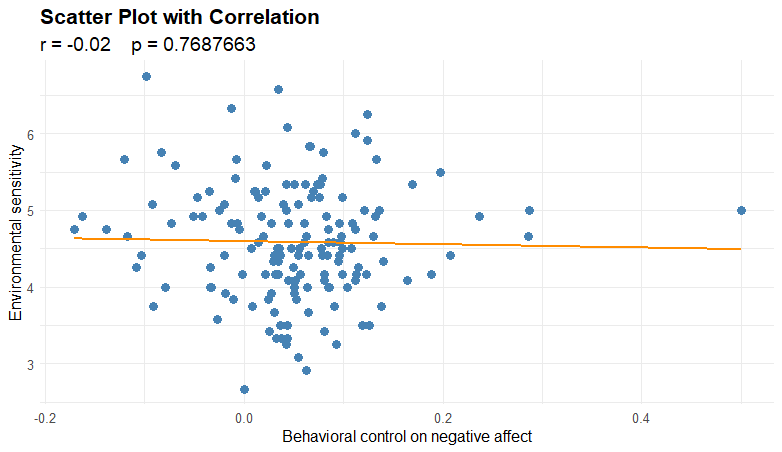


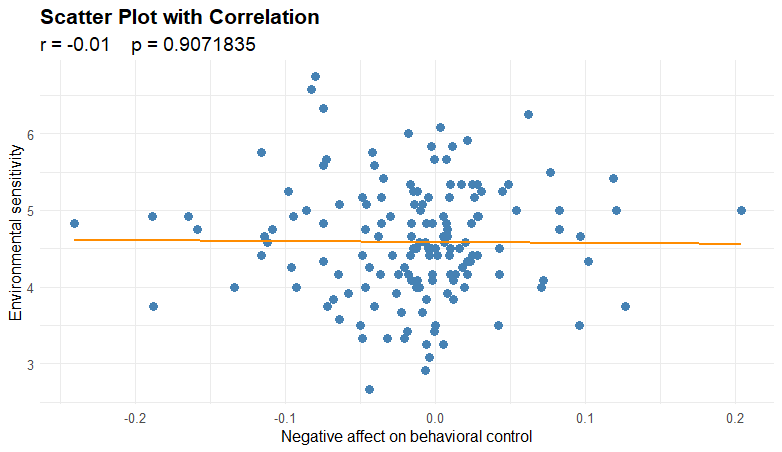


**Figure S6**

*Correlations of Environmental Sensitivity with the Family-Specific Day-to-Day Effects between Parental Autonomy Support and Adolescent Positive Affect*


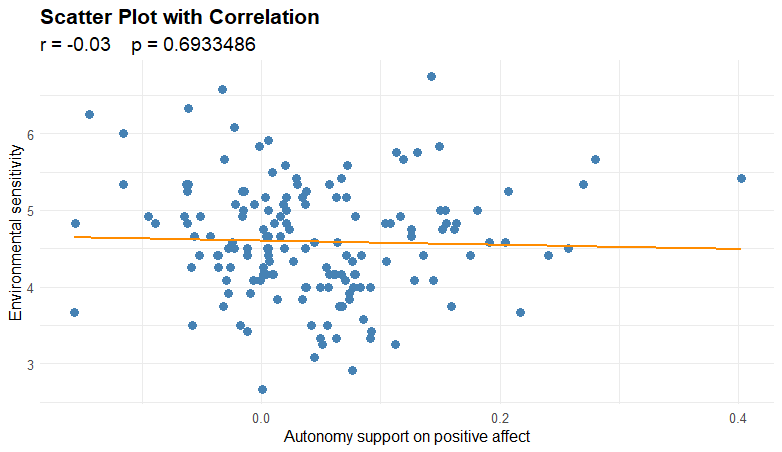


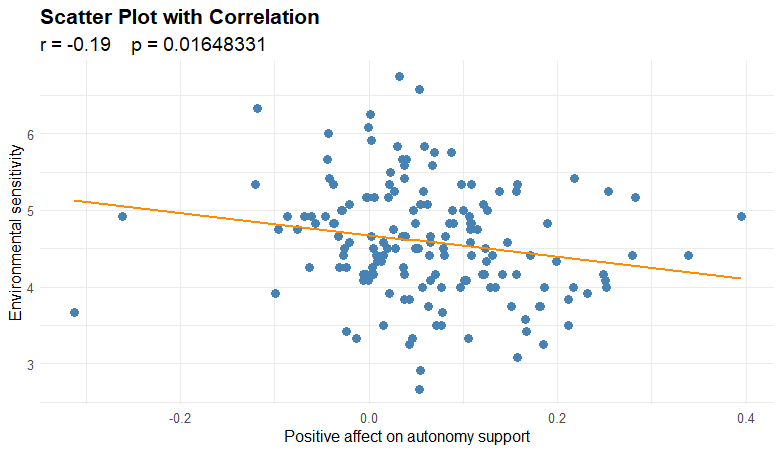


**Figure S7**

*Correlations of Environmental Sensitivity with the Family-Specific Day-to-Day Effects between Parental Autonomy Support and Adolescent Negative Affect*


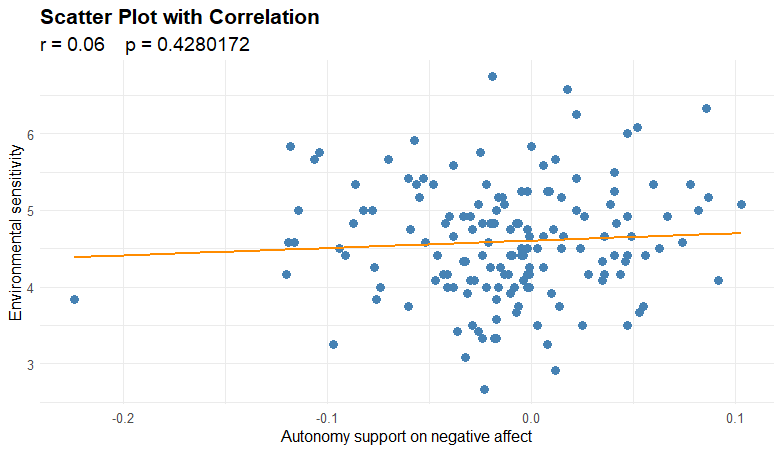


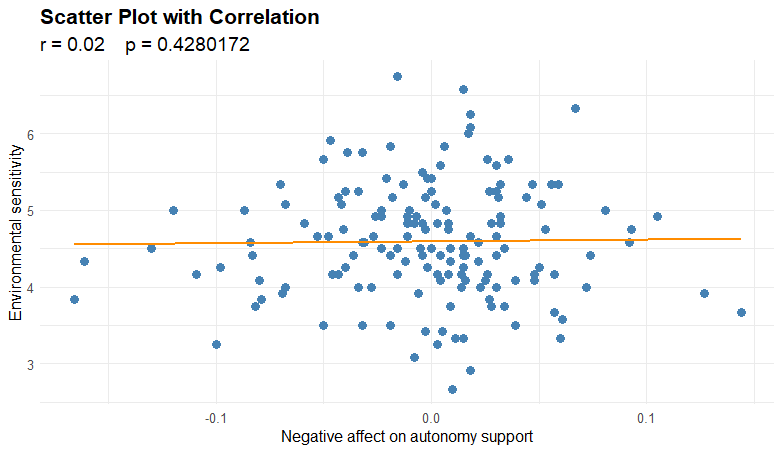


**Figure S8**

*Correlations of Environmental Sensitivity with the Family-Specific Day-to-Day Effects between Parental Warmth and Adolescent Positive Affect*


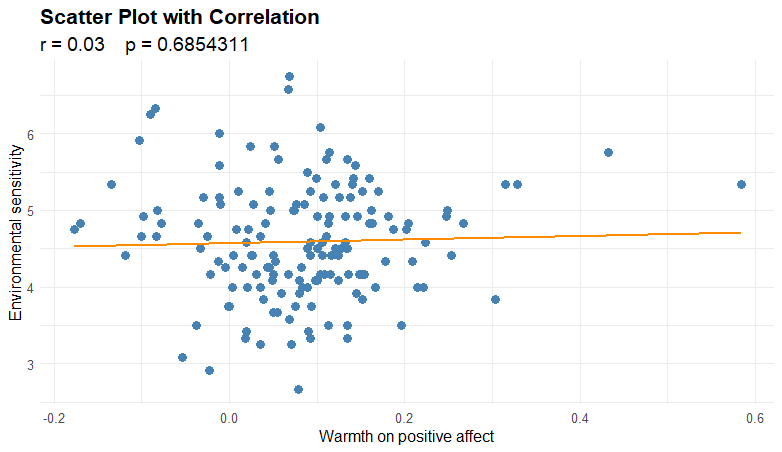


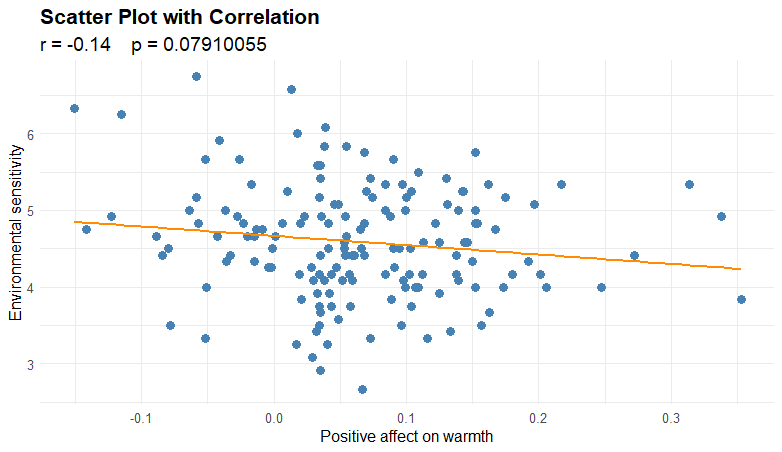


**Figure S9**

*Correlations of Environmental Sensitivity with the Family-Specific Day-to-Day Effects between Parental Warmth and Adolescent Negative Affect*


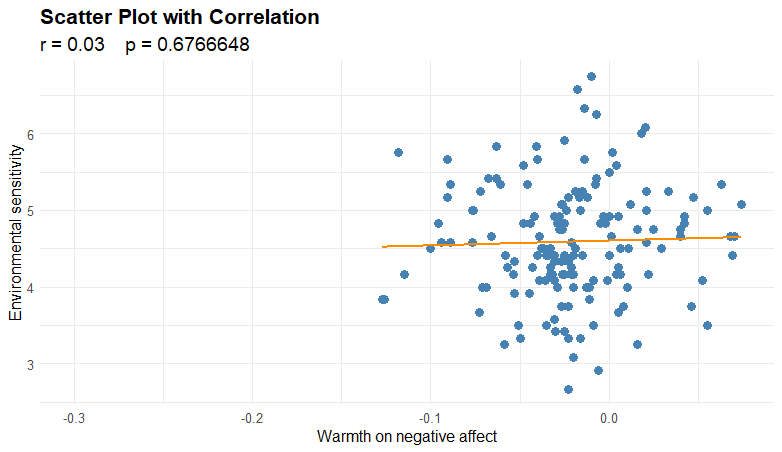


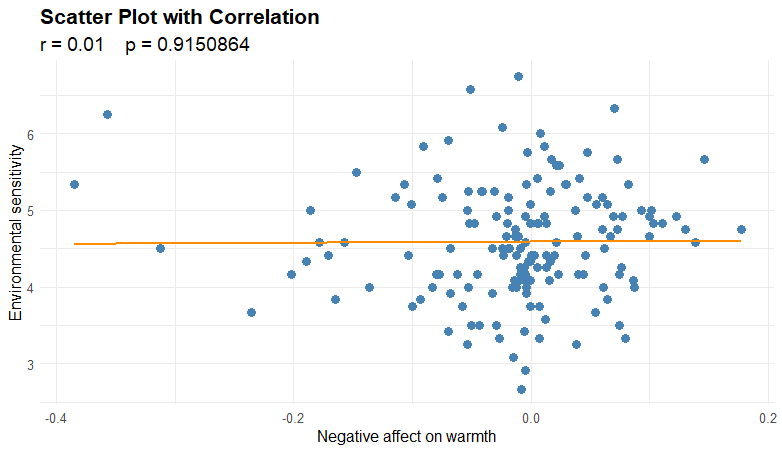

Supplement: Supplementary file 1 — Supplementary Information. [file 41598_2023_43294_MOESM1_ESM.docx]
